# Relationships Between the Yo-Yo Intermittent Recovery Test and Anaerobic Performance Tests in Adolescent Handball Players

**DOI:** 10.1515/hukin-2015-0020

**Published:** 2015-04-07

**Authors:** Souhail Hermassi, Ridha Aouadi, Riadh Khalifa, Roland van den Tillaar, Roy J. Shephard, Mohamed Souhaiel Chelly

**Affiliations:** 1 Higher Institute of Sport and Physical Education of Ksar Said, Manouba University, Tunis, Tunisia.; 2 Research Unit « Sport performance & health.; 3 Department of Teacher Education of Nord Trøndelag University College, Norway.; 4 Faculty of Physical Education & Health, University of Toronto, Toronto, Canada.

**Keywords:** handball, lower limb muscular power, jumping performance, force–velocity test, sprint velocities

## Abstract

The aim of the present study was to investigate relationships between a performance index derived from the Yo-Yo Intermittent Recovery Test level 1 (Yo-Yo IR1) and other measures of physical performance and skill in handball players. The other measures considered included peak muscular power of the lower limbs (Wpeak), jumping ability (squat and counter-movement jumps (SJ, CMJ), a handball skill test and the average sprinting velocities over the first step (VS) and the first 5 m (V5m). Test scores for 25 male national-level adolescent players (age: 17.2 ± 0.7 years) averaged 4.83 ± 0.34 m·s^−1^ (maximal velocity reached at the Yo-Yo IR1); 917 ± 105 Watt, 12.7 ± 3 W·kg^−1^ (Wpeak); 3.41 ± 0.5 m·s^−1^ and 6.03 ± 0.6 m·s^−1^ (sprint velocities for Vs and V5m respectively) and 10.3 ± 1 s (handball skill test). Yo-Yo IR1 test scores showed statistically significant correlations with all of the variables examined: Wpeak (W and W·kg^−1^) r = 0.80 and 0.65, respectively, p≤0.001); sprinting velocities (r = 0.73 and 0.71 for VS and V5m respectively; p≤0.001); jumping performance (SJ: r = 0.60, p≤0.001; CMJ: r= 0.66, p≤0.001) and the handball skill test (r = 0.71; p≤0.001). We concluded that the Yo-Yo test score showed a sufficient correlation with other potential means of assessing handball players, and that intra-individual changes of Yo-Yo IR1 score could provide a useful composite index of the response to training or rehabilitation, although correlations lack sufficient precision to help in players’ selection.

## Introduction

Competitive handball is an intensive form of intermittent physical activity that requires optimal development of both aerobic and anaerobic capacity (Hermassi et al., 2011; Massuca et al., 2013). Performance is related more to velocity, agility, strength, explosive power, and the ability to repeat brief supra-maximal bouts of exercise than to either ball-handling skills or the capacity for sustained sub-maximal mechanical work (Hermassi et al., 2011; Ingebrigtsen et al., 2013; Povoas et al., 2012; Souhail et al., 2010). Monitoring of the development of appropriate physical characteristics during training is usually based upon a battery of specific tests that mimic the activities inherent to the game (Ingebrigtsen et al., 2013; Souhail et al., 2010; Buchheit et al., 2012; Jensen et al., 1997), and reproduce the technical skills, movement patterns, and physiological demands of competitive matches (Bangsbo et al., 1994; Bangsbo et al., 1992; Buchheit et al., 2009; Povoas et al., 2012).

Handball play is characterized by repeated periods of intense anaerobic activity (Kruger et al., 2013; Manchado et al., 2013), and it is thus logical to evaluate a player’s overall ability in terms of his or her tolerance of repeated bouts of intensive exercise. The Yo-Yo Intermittent Recovery Test Level 1 (Yo-Yo IR1), as proposed by Bangsbo (1991), seems well-suited to this purpose. It requires repeated bouts of effort at progressively increasing velocities, interspersed with 10 s intervals of active recovery, with the test continuing to exhaustion. The test has been validated both externally and internally (Souhail et al., 2010; Bangsbo et al., 2008; Krustrup and Bangsbo, 2001; Krustrup et al., 2003); muscle biopsy and blood analyses have demonstrated that it elicits maximal aerobic responses at the same time as it imposes a significant stress upon the anaerobic energy system (Krustrup et al., 2003).

The Yo-Yo IR1 test has achieved significant popularity in both research and practical settings (Bangsbo et al., 2008; Buchheit and Rabbani, 2013; Delahunt et al., 2013). It has proven a reliable and valid measurement of match-related fitness in youth handball players (Souhail et al., 2010), demonstrating a capability to distinguish differences of a player’s performance at various levels of competition, in diverse playing positions, and after varying periods of training (Bangsbo et al., 2008). Further, the fatigue caused by performance of the Yo-Yo IR1 test is rather similar to the fatigue that develops and causes a deterioration of skills over the course of an experimental soccer game (Impellizzeri et al., 2008; Rampinini et al., 2010). Based on these results, the Yo-Yo IR1 test is now considered the single most relevant field performance test for the evaluation of adult soccer, basketball and rugby players (Atkins, 2006; Castagna et al., 2008).

However, information about the sensitivity of the Yo-Yo IR1 test and its relationship to other commonly used measures of athletic performance remains quite limited. Heart rates have been measured during testing (Souhail et al., 2010; Krustrup et al., 2003; Castagna et al., 2008), but relationships between the Yo-Yo IR1 scores and other field measures of anaerobic performance have yet to be examined. Thus, we examined relationships between Yo-Yo test performance and the peak muscular power of the lower limbs, jumping and sprinting abilities and a handball skill test. It was hypothesized that the Yo-Yo IR1 test would provide a simple inclusive index of a person’s capacity for handball play, indicating not only aerobic qualities and the ability to recover between exercise bouts, but also strength and maximal explosive power of the lower limbs.

## Material and Methods

Our objective was to assess the extent of associations between the maximal intermittent running velocity reached at the end of the Yo-Yo IR1 test (the Yo-Yo IR1 score) and other characteristics of a successful handball player [explosive power of the lower limbs as estimated by a force-velocity test, squat (SJ) and counter movement (CMJ) jump scores, the velocities reached in the first step after a sprint start (V_S_) and over 5-m (V_5_m), and ball throwing velocity]. All participants completed two familiarisation sessions over the two weeks prior to definitive testing. On each occasion, the protocol included the Yo-Yo IR1 test, a cycle ergometer force-velocity test of lower limb muscular power, the SJ and CMJ, sprint performance and a handball skill test (a measure of speed, agility, and skill as reflected in slalom dribbling ability). All observations were made at the same time of the day, and under the same experimental conditions, at least 3 days after the most recent competition. Subjects maintained their normal intake of food and fluids. They abstained from physical exercise for one day, drank no caffeine-containing beverages for four hours, and ate no food for two hours. Verbal encouragement ensured maximal effort throughout testing.

### Participants

Twenty five male youth handball players (age: 17.2 ± 0.7 years; body mass: 88.7 ± 6.1 kg; body height: 1.87 ± 0.86 m; percentage body fat: 13.3 ± 1.4%; handball experience: 8.3 ± 0.2 years) were recruited from a single national-level team under conditions approved by the Manouba University Committee on Human Experimentation. Participants were fully informed about the protocol before the start of the study. Informed consent was obtained from all players and their parents prior to testing, in accordance with the recommendations of local ethical committee and current ethical standards in sports and exercise research. All of those tested were also given a preliminary examination by the team physician, who focused on orthopedic and other conditions that might preclude testing or impair performance, and found all were in good health.

### Measures

All assessments were performed at the same time of the day (from 5.00 to 7.00 p.m.) and subjects were blinded as to the specific aims of the study. Testing was carried out during the competitive season, two months after the beginning of the national championships; the selected players were taking part in national and international championships at the time of the investigation. They thus continued to undertake 90 min of handball training three to four times per week; these sessions aimed at enhancing skill activities at various intensities, offensive and defensive tactics, and included 30 min of continuous play with only brief interruptions. They also played one official handball game per week, and engaged in 40 min of physical education per week that comprised mainly ball games. Tests were completed in a fixed order, over three consecutive days.

Body mass was measured to the nearest 0.1 kg, using an electronic scale (Seca, Hamburg, Germany) and standing height was measured using a wall stadiometer (Tanita, Ancona, Italy). Body fat content was assessed as described by Womersley (Womersley and Durnin, 1973); all skinfold measurements (Harpenden caliper, Basty International, Burgess Hill, UK) were made by the same experienced examiner, and body fat content was expressed as a percentage of body mass.

### Procedures Day 1

#### Yo-Yo Intermittent Recovery test Level 1 (Yo-Yo IR1)

The Yo-Yo IR1 test (MIRV) was performed according to the procedures suggested by Krustrup (2003) and Castagna (2008). Test reliability was established in a previous study (Krustrup et al., 2003). A short-range telemetric heart rate monitor (S 810, Polar Electro Oy, Kempele, Finland) was placed on the player approximately 20 min before testing. The heart rate was monitored throughout the test, using a 5 s interval recording time. Post-hoc HR analyses were performed using Polar Precision System SW software (Polar Electro Oy, Kempele, Finland). The peak recorded HR was assumed to be the individual’s maximal HR (Krustrup et al., 2003).

### Day 2

#### Force–velocity test

A force-velocity test was performed on a standard Monark cycle ergometer (model 894 E, Monark Exercise AB, Vansbro, Sweden), as detailed elsewhere (Chelly et al., 2009; 2010). In brief, the maximal pedalling velocity attained during a 7 s all-out sprint was used to calculate the maximal anaerobic power for each braking force, and the highest peak leg power (Wpeak) was reached if a further increase of loading induced a decrease in power output.

### Day 3

#### Squat Jump (SJ) and Countermovement Jump (CMJ)

Characteristics of the SJ and the CMJ were determined using a force platform (Quattro Jump, version 1.04; Kistler Instrumente AG, Winterthur, Switzerland). Jump height was determined as the centre of mass displacement, calculated from the recorded force and body mass. Subjects were instructed to keep their legs straight throughout the flight phase. The SJ began at 90° knee flexion; a vertical jump was performed by pushing upward with the legs, avoiding any downward movement. The CMJ began from an upright position; subjects made a downward movement to 90° knee flexion and simultaneously began the push-off phase. The best of 3 jumps was recorded for each test.

#### Sprint Performance

After a familiarization session with the sprint test, subjects performed a maximal 5 m sprint on an outdoor tartan surface. Body displacement was filmed by a camera (Sony Handycam, DCR-PC105E; 25 frames per second, Tokyo, Japan) placed at the 5 m mark, perpendicular to the running lane. Two trials were separated by an interval of at least 5 min, with the fastest time being recorded. The software (Regavi & Regressi; Micrelec, Coulommiers, France) converted measurements of hip displacement to velocities reached during the first step (V_S_) and over the first 5 m (V_5_m). The reliability of the camera and data processing software has been described previously (Chelly et al., 2009). We chose a standing start to give greater consistency to our measurements, although recognizing that in actual play a sprint usually begins from a standing or jogging start.

### Day 4

#### Handball skill test

Speed, agility, and handball skills were tested by a slalom dribbling test. Subjects ran a distance of 15 m, back and forth, dribbling a handball around 5 cones. The distance between the starting line and the first cone, as well as between the other cones, was 3 m. Subjects ran individually (Burgess et al., 2010). The better of 2 trials was recorded for statistical analysis. All tests were performed on an indoor synthetic pitch, and electronic timing gates (Microgate Racetime 2 Light Radio, Bolzano, Italy) were used to record times.

### Statistical Analysis

Findings are reported as means ± standard deviations (SD). The reliability of jump tests (SJ and CMJ), track running velocity and the handball skill test were assessed using intraclass correlation coefficients (ICC). Pearson’s product moment correlations and linear regression analyses were used to examine relationships between the Yo-Yo IR1 test performance and other measures of physical ability. Significance was assumed at 5% (p≤ 0.05). All statistical analyses were performed using the Statistical Package for the Social Sciences (SPSS) (version 19.0 software for windows).

## Results

**Figure 1:**
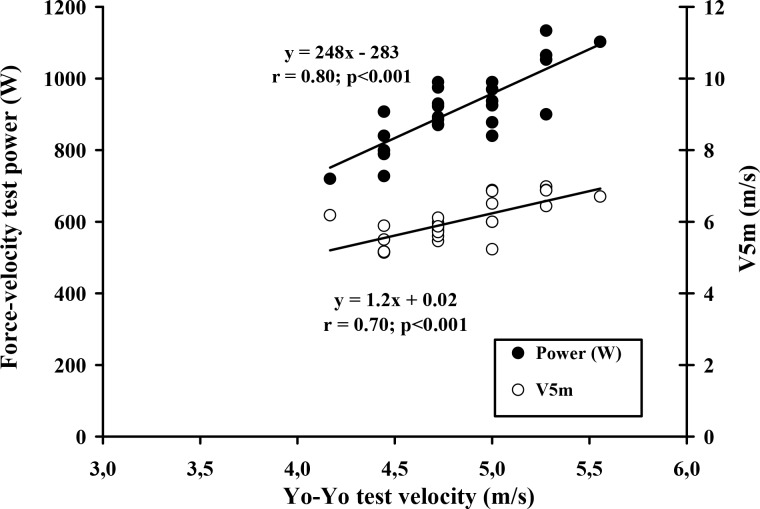
Strong relationships between maximal velocity reached in the Yo-Yo intermittent recovery test and both leg peak power and 5 m sprint performance

**Figure 2:**
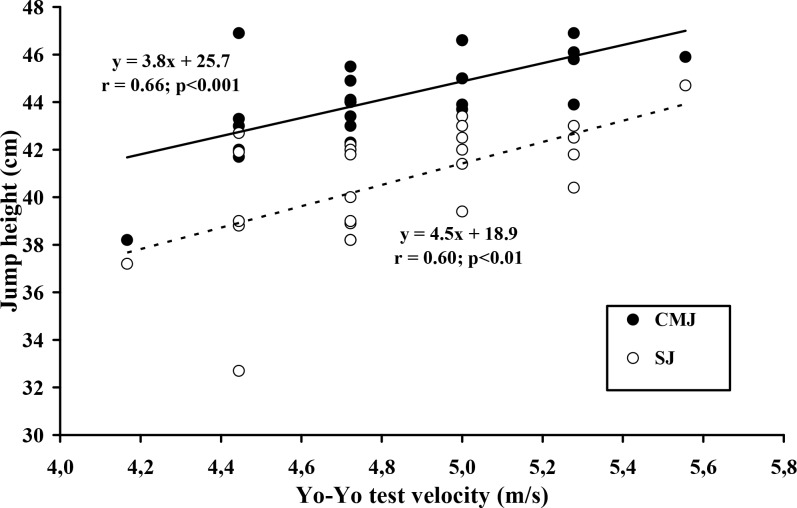
Strong relationships between maximal velocity reached in the Yo-Yo intermittent recovery test and both jump height: Counter Movement Jump and Squat Jump (CMJ and SJ, respectively)

The ICCs for SJ (0.92), CMJ (0.81), velocity for the first step after starting to run (0.88), velocity over the first 5 m (0.91) and the handball skills test (0.96) were all at levels rated as moderate (between 0.80 and 0.90) or high (more than 0.90) for physiological field tests. The total distance covered during the Yo-Yo IR1 test averaged 1772 ± 343 m, the maximal intermittent running velocity (MIRV) was 17.4 ± 1.2 km·h^−1^ and the peak heart rate averaged 199.9 ± 1.7 beat·min^−1^. Values for absolute and relative force-velocity test measures of leg power (Wpeak), V_S_ and the V_5m_ were 917 ± 105 Watts, 12.8 ± 2.3 W·kg^−1^, 3.41 ± 0.51 m·s^−1^ and 6.03 ± 0.6 m·s^−1^, respectively (Table 1). The MIRV was significantly correlated with almost all other measured parameters (Table 2), the largest correlation coefficients (r = 0.80 and r = 0.71; p≤0.001) being with the absolute Wpeak for the lower limbs (Figure 1) and with the skill test, V_S_ and V_5_m (r = 0.73 and 0.70, respectively; p≤0.001) and the lowest with the jump tests (SJ and CMJ, 0.60 and 0.66; Figure 2).

## Discussion

In accordance with our hypothesis, the main finding of this study was that performance of the Yo-Yo IR1 test provided an overall assessment of the handball player, the maximal intermittent running velocity on this test showing significant and substantial correlations with scores for several of the other tests commonly used to assess explosive force and skills in this sport. In the present paper, we will comment briefly on each of these associations.

### Performance of the Yo-Yo IR1 test and power of the lower limbs

The Yo-Yo IR1 test focuses both on the capacity to carry out intermittent exercise, leading to maximal activation of the aerobic system, and the individual’s ability to recover from repeated exercise, with a high contribution from the anaerobic system (Krustrup et al., 2003). Recently Karakoc et al. (2012) found only moderate relationships between peak power and the fatigue index in a Wingate test and the Yo-Yo IR2 performance, but the association observed in their investigation was likely weakened by a difference in the mode of exercise (i.e. cycle ergometry vs. running). Moreover, handball players are more familiar with running than with cycling, and lower limb cranking is essentially a cyclic movement, whereas sprinting reflects the power obtained from a single whole-body movement (Wadley and Le Rossignol, 1993; Wragg et al., 2000). Despite the difference in test modality, substantial correlations were seen between the MIRV and the peak power output in our 7 s maximum cycle ergometry test. Possibly, our cycle ergometer protocol approximated more closely to the muscle dynamics and contraction times associated with the sprinting tests.

High levels of neuronal activation are needed to reach maximal sprint velocities. Potential mechanisms developing sprint performance in handball players include more efficient movement due to changes in temporal sequencing of muscle activation, preferential recruitment of fast motor units and increased nerve conduction velocity (Wragg et al., 2000). Our results demonstrate that subjects with a high cycle ergometer power output are superior sprinters (Wragg et al., 2000).

### Jumping performance

Competitive handball requires frequent turning and changes of directions at a variety of intensities (Hermassi et al., 2011), and this demands both muscular power and strength. The vertical jump performance of handball players varies with their competitive level (Hermassi et al., 2011; Gorostiaga et al., 2006), showing that this field test provides a useful measure of their ability. Again, scores on this test are significantly correlated with the Yo-Yo MIRV. Castagna et al. (2006) also found a significant correlation between the MIRV and vertical jump performance. We expected that these two measures would be related because of the similarities in the muscle contraction pattern (stretch shortening cycle); many other results in the scientific literature show moderately close relationships between sprint and jumping performance.

### Relationship between Yo-Yo IR1 and sprint velocities

The initial sprint velocity and acceleration over the first few seconds of running are important data for coaches, and the ability to accelerate over a single step is a critical factor in some game situations (Chelly et al., 2010). However, regular filming of handball players is hardly practical for most teams. We noted a strong positive relationship between the velocity over the first step and the MIRV. During this first step, the foot tread is far ahead of the hips. This creates a significant variation from the “pushing” position of an acceleration movement (Mann, 1994). An athlete who placed his foot in this position while trying to accelerate would stand upright prematurely, taking him or herself out of the optimal position for acceleration. The maximum incline should be around 3 degrees; because beyond this angle, sprinting is negatively affected (Mann, 1994).

Maximizing of velocity over the first 5 m is a complex motor task, characterized by the ability to develop large forces in the horizontal plane over a short time period (Hafez et al., 1988). The relationship of the V_5_m to the MIRV has not been investigated previously. The existence of strong relationships is at first inspection somewhat surprising, since the two tests were intended to measure apparently differing abilities (endurance and muscle explosive force). However, the Yo-Yo IR1 test requires a sprint start with each change of direction, which could explain at least some of the observed relationship.

### Handball skill test

The strong relationship between the MIRV and the handball skill test is likewise somewhat surprising, since the items purportedly measured are endurance vs. agility and speed. As in the sprint test, the skill test is essentially based on sprinting forward or backwards, with the subject exerting explosive force at each change of direction. In the slalom dribbling skill test used in this study, the player is instructed to adopt only a single pattern of movement (dribbling back and forth) in order to cover the distance as rapidly as possible. One possible factor contributing to the positive correlation between the Yo-Yo IR 1 test and dribble test could be the acceleration and deceleration of the legs and changes of direction during the turns in Yo-Yo IR 1 test. Possibly, this demands similar abilities to the dribbling test.

The present study suggests that much of the information obtained from a substantial battery of field performance tests could be obtained more simply by having players undertake only the Yo-Yo IR1 test. Scores on this test accounted for 50–60% of the variance in the individual test measurements. If some of the existing tests were to be eliminated, this would allow coaches the opportunity to focus more on the assessment of other skills, such as throwing abilities. However, the high correlations must be interpreted with caution, since they could reflect in part sources of covariance, particularly those associated with inter-individual differences in body build; this criticism is supported by the attenuation of the peak power correlation when data are expressed per kg of body mass.

An obvious next step in this analysis will be to evaluate how far gains in the MIRV can predict gains in individual test scores, as players undergo a rigorous training program. It will also be important to extend observations to players in other age groups (including females), and at other sports levels. It will also be of interest to correlate observations of this type with physiological estimates of anaerobic tolerance such as anaerobic thresholds and blood lactate levels. Finally, it may be interesting to compare findings on the dribbling tests with the scores observed when performing the same movement patterns, yet with the ball.

The present results, obtained on top-level youth handball players, point to the conclusion that the performance of a single field test, the MIRV on the Yo-Yo IR1 test, provides information that is strongly correlated with the results obtained from a series of field performance tests for team athletes. It requires a minimum of equipment, and may thus prove an effective measure of the response of individual players to both training and rehabilitation.

## Figures and Tables

**Table 1:** **Mean ± SD of the parameters measured**

	
Test	Mean ± SD (n=25)
*Yo-Yo Intermittent recovery test*	
Maximal running velocity (m/s)	4.8 ± 0.3
Total distance covered (m)	1772 ± 343
Time spent (min)	15.0 ± 2.5
*Force-velocity test*	
Peak power (W)	917 ± 105
Peak power (W/kg)	12.7 ± 2.3
*Jump test*	
Squat jump height (m)	0.40 ± 0.2
Countermovement jump height (m)	0.44 ± 0.1
*Sprint test*	
Velocity after first step (m/s)	3.4 ± 0.5
Velocity after 5m (m/s)	6.0 ± 0.6
*Handball skill test*	
Slalom dribbling test (m/s)	10.3 ± 1

**Table 2:** Correlations between the maximal running velocity during the Yo-Yo IR1 test and scores for other test parameters (n=25)

	
	Parameter	Correlation with Yo-Yo maximal running velocity
Force-velocity test	Peak power (W)	0.80^***^
	Peak power (W/kg)	0.65^***^
Jump test	Squat jump height (m)	0.60^**^
	Counter movement jump height (m)	0.66^***^
Sprint test	Velocity after first step (m/s)	0.73^***^
	5-m sprint (m/s)	0.71^***^
Slalom dribbling test	Slalom dribbling test (m/s)	0.71^***^

** Significant correlation with the maximal running velocity from the Yo-Yo IR1 test at p≤0.01 level
